# Safeguarding vaccine production and supply strategies for polio eradication endgame

**DOI:** 10.1016/j.lanwpc.2021.100159

**Published:** 2021-05-12

**Authors:** Ananda S. Bandyopadhyay, Miguel O'Ryan

**Affiliations:** a Polio, Global Development, Bill & Melinda Gates Foundation, Seattle, USA; b Microbiology and Mycology Program, Institute of Biomedical Sciences, Faculty of Medicine, University of Chile, Chile; c Millennium Institute of Immunology and Immunotherapy, Faculty of Medicine, University of Chile, Chile

The world is tantalizingly close to eradicate the second human disease in the history of global health. Compared to late 1980s when the Global Polio Eradication Initiative (GPEI) was launched, incidence of wild poliovirus (WPV) cases has been reduced by more than 99.9%, two of three serotypes of WPV have been certified eradicated and all but two countries – Pakistan and Afghanistan – have successfully interrupted endemic WPV transmission. However, an uptick of WPV type 1 cases in these two endemic countries in the recent past combined with a rise in circulating vaccine-derived poliovirus (cVDPV) spreading across more than twenty countries in Africa have proved to be issues of major concern in the final phase of the eradication battle [1].

In addition to overcoming the evolving challenges of persistent WPV type 1 circulation in the two endemic countries and the expansion of cVDPV circulation in the African continent, safeguarding post-eradication risks of re-introduction of poliovirus transmission is a key strategic focus of the GPEI. In the post-eradication era, accidental leakage of WPV from vaccine manufacturing plants could lead to community exposure and paralytic outbreaks. Applying containment strategies and maintaining high population immunity through routine immunization with inactivated poliovirus vaccine (IPV) are expected to minimize such risks and the fallout [2]. In addition, producing IPV from less infectious sources such as the Sabin strains compared to the current practice of using wild strains is yet another promising strategy to enhance biosafety and thereby protecting the progress made on the eradication front [3].

Sabin strain IPV (sIPV) formulations have been in development for almost a decade, with on-going use in China and Japan [4]. Major progress has been made to establish international standards to measure the potency of Sabin IPV candidates *in vitro* in the recent past. Also, in December 2020, Sabin IPV manufactured at LG Chem received WHO Prequalification paving the way for boosting IPV supplies in low and middle income countries [5]. With this backdrop of on-going progress and promise with Sabin IPV as a tool for the Endgame of polio, the study from Zhijie An and colleagues in this issue provides novel evidence base for a dose-sparing strategy [6]. In a simple open-label, two *vs* three dose regimen trial, study participants were randomized to receive either 3 doses of sIPV at 2,3 4 months, or 2 doses at 4 months and at 8–11 months. Seroprotection rates achieved one month after the last vaccination, 5 months in the 3 dose and 9–12 months in the 2 dose groups, was greater than 98% for all three polio serotypes in both schedules while median titers were significantly higher in the 2 dose group for all serotypes. Enhanced immunogenicity of the delayed 2-dose regimen with longer interval between doses is consistent with our understanding of impact of maternally-derived antibodies on IPV when administered early in infancy.

These results should encourage similar studies with other Sabin IPV vaccine candidates in infant immunization schedules with less than 3 doses. The tradeoffs across delayed, dose sparing schedules with cost and supply advantages have to be carefully balanced out with any potential risk of vulnerability of young infants from on-going polio transmission in high-risk areas. Non-inferiority of delayed, SAGE recommended schedules of Salk IPV – either in full or fractional doses – have also been demonstrated in randomized controlled trials [7]. Thus, the data presented by Zhijie An and colleagues help generate a comprehensive evidence base around novel schedules with different types of IPV formulations that are particularly relevant for policy formulation for immunization schedules in the post-OPV cessation era.

All strategies aiming to improve biosafety measures of polio vaccine production with the promise of further cost saving through dose sparing are important to strengthen the chances of success to complete and maintain polio eradication. This study is a valuable addition to the evidence base around the option of reduced number of sIPV doses infant inmunization schedules and should therefore expand the supply possibilities of IPV produced from safer sources in the final phase of polio eradication and beyond.

## Declaration of Competing Interest

We declare no competing interests.
